# Reviews

**Published:** 1890-11

**Authors:** 


### Book Reviews.

---

ESSENTIALS OF ANATOMY and Manual of Practical Dissection, together with the Anatomy of the Viscera. Prepared Especially for Students of Medicine. By Charles B. Nancrede, M. D., Professor of Surgery and of Clinical Surgery in the University of Michigan, Ann Arbor, etc. Third edition; revised and enlarged. Based upon the last edition of Gray's Anatomy. Thirty handsome full-page lithographic plates, in colors, and 180 fine wood-cuts. Post 8vo; pp. x.—388. Philadelphia: W. B. Saunders. 1890.

The author undertook to embody in a concise and judicious way, in this small work, the essentials of anatomy, and he has succeeded most admirably. He has culled from the larger works, and especially from Gray, just such anatomical knowledge as the practitioner is most apt to need. The definitions are in almost every instance complete, so that the student gets just such distinctive facts, with the structures mentioned, as will fix them in his memory. We do not see how the student can be injured by this compend. It is intermediate between the ordinary compend and a larger work upon anatomy, and thus avoids the bad features of the former while possessing many of the advantages of the latter. We, therefore, recommend it to the student and practitioner.

The plates are beautiful and clear, the type excellent, and the whole supplemented with a full index. The author and publishers have done the profession a service in bringing out this admirable little book. J. P.

---

WOOD'S MEDICAL AND SURGICAL MONOGRAPHS, Consisting of Original Treatises and Reproductions in English of Books and Monographs selected from the latest literature of foreign countries, with all illustrations, etc. Published monthly. Vol. VII., No. 1, July, 1890. No. 2, August, 1890. No. 3, September, 1890. New York: William Wood & Company. $10.00 a year; single copies, $1.00.

In the number of this valuable series for July, 1890, may be found the following interesting and instructive articles: Stricture of the Rectum, by Charles B. Kelsey, M. D.; The Influence of Heredity on Alcoholism, by Paul Sollier; Rabies, by Louis Pasteur, Paris; Colotomy, Lumbar and Iliac, with Special Reference to the Choice of Operation, by Thomas Bryant, F. R. C. S.; Massage of the Abdomen, by Rubens Hirschberg, M. D.

In the number for August, 1890, the following subjects are treated: Morbid Blushing, its Pathology and Treatment, by Harry

Campbell, M. D.; Alcoholism in Women, by Dr. Thomeuf; The Different Methods of Lifting and Carrying the Sick and Injured, by G. H. Darwin, M. D.; Treatment of Ingrowing Toe-Nail, by Joseph Amiard, M. D.; Chronic Bronchitis and its Treatment, by William Murrell, M. D.

The number for September, 1890, is devoted to the consideration of Insomnia and its Therapeutics, by A. M. Macfarlane, M. D. An index for these three numbers accompanies the last one, which makes a complete volume for binding.

We have heretofore frequently expressed our opinion of the value of the monographs that this great publishing house are putting forth in this form, and these three numbers are fully up to the standard. The most of the titles they contain are of interest to the general practitioner, and a few to the surgeon, while all must make haste to possess this last one, that every medical man will find instructive.

